# A New Estimator of Kullback–Leibler Divergence via Shannon Entropy

**DOI:** 10.3390/e28070720

**Published:** 2026-06-24

**Authors:** Mehmet Sıddık Çadırcı, Martin Singull

**Affiliations:** 1 Department of Statistics, Faculty of Science, Cumhuriyet University, 58140 Sivas, Türkiye; msiddikcadirci@cumhuriyet.edu.tr; 2 Department of Mathematics, Linköping University, 581 83 Linköping, Sweden

**Keywords:** Kullback–Leibler divergence, Shannon entropy, kNN estimation, goodness-of-fit test, multivariate normality

## Abstract

We examine the estimation of the Kullback–Leibler (KL) divergence and the use of the goodness-of-fit test for multivariate normality. Our starting point is the maximum entropy principle for Shannon entropy: among all distributions with a fixed mean vector and covariance matrix, the multivariate Gaussian distributions uniquely maximize entropy. As a result, the KL divergence from a moment-matched Gaussian distribution to an unknown density can then be written as the entropy difference, which is a suitable information-theoretic measure of divergence from the Gaussian distribution. To estimate, we use *k*-nearest neighbor (kNN) estimators based on Shannon entropy and KL divergence derived from the Kozachenko–Leonenko approach and subsequent improvements, along with the consistency and $L^{2}$-convergence results established for these estimators. Motivated by previous entropy-based goodness-of-fit ideas developed for Rényi-type functionals for generalized Gaussian and Student-type models, we describe a KL-based test statistic as being the difference between the entropy of a Gaussian model fitted to the sample mean and covariance and the KL divergence between the unknown entropy and the kNN estimate. The statistic converges to zero for multivariate normality and converges to a strictly positive bound with non-Gaussian alternatives. The results of Monte Carlo simulations conducted across various dimensions and sample sizes indicate that the proposed method provides accurate Type I error control among the alternatives considered and demonstrates promising empirical power.

## 1. Introduction

The Kullback–Leibler (KL) divergence, known also as relative entropy, is a fundamental measure in information theory and statistics used to quantify the difference between two probability distributions. It was introduced by Kullback and Leibler [1] and measures the expected log-likelihood loss (or excess log-loss) that arises when a reference model *g* is employed instead of the true distribution *f*. Because of this decision-theoretic interpretation, KL divergence occurs naturally in probability-based inference, model selection, goodness-of-fit testing, density comparison, and a wide variety of learning and signal processing tasks.

The KL divergence from *f* to *g* is defined as follows for two continuous probability density functions (pdf) $f,g:R^{m}\to R$:

(1) $$D_{KL}\left(f∥g\right)=\int_{R^{m}}f\left(x\right)\log\frac{f(x)}{g(x)}\;dx.$$

Since the divergence $D_{KL}\left(f∥g\right)\geq 0$ and equals zero only when f=g almost everywhere, it measures the difference between distributions in a “directional” manner despite not being symmetric and thus not being a metric.

The KL divergence is closely related to Shannon entropy, measuring the uncertainty in a single distribution. The differential Shannon entropy H(f) [2] for density *f* is given by

(2) $$H\left(f\right)=-\int_{R^{m}}f\left(x\right)\log f\left(x\right)\;dx,$$

and a simple processing yields the following result

(3) $$D_{KL}\left(f∥g\right)=-H\left(f\right)-\int_{R^{m}}f\left(x\right)\log g\left(x\right)\;dx.$$

Equivalently, $D_{KL}\left(f∥g\right)$ is defined as the cross-entropy $-E_{f}\left[\log g\left(X\right)\right]$ minus the entropy H(f) and thus can be considered as the excess cross-entropy (or excess expected log loss) that is associated with using the oracle model *g* in place of *f*.

Equation (3) also clarifies why certain comparison distributions are particularly natural in KL-based procedures. Under constraints of fixed mean and covariance, the multivariate Gaussian distribution maximizes Shannon entropy; see, e.g., [3,4,5,6]. Therefore, among all distributions that share the same first and second moments, the Gaussian is the only distribution that maximizes entropy. Similarly, $D_{KL}\left(f∥\phi_{\mu ,\Sigma }\right)$ is the difference in entropy $H\left(\phi_{\mu ,\Sigma }\right)-H\left(f\right)$ and gives a principled measure of deviation from the Gaussian distribution where $\phi_{\mu ,\Sigma }$ is the adapted Gaussian distribution matching the moments of *f*. The observation motivates Gaussian benchmarks in goodness-of-fit tests and forms the basis for the KL-based procedures developed in this work.

Estimating the value of $\left(D_{KL}\left(f|g\right)\right)$ from data is of vital importance in practical applications such as hypothesis testing, density comparison, and anomaly detection. Classical add-on approaches using histograms or kernel density estimators have been successful in low-dimensional settings, but as the dimension (m) increases with respect to the sample size (N), its accuracy may become sensitive [7,8,9] to the choice of bin width or bandwidth and the sparsity of local observations. Here, instability refers to the increasing variability and sensitivity to tuning parameters of the obtained density or variance estimates. In the numerical experiments below, our focus is on dimensions up to (m=3), which should be interpreted as a medium-dimensional multivariate setting instead of a truly high-dimensional regime. Nearest-neighbor (NN) methods provide an appealing alternative: these methods employ the local geometric structure of the sample cloud instead of explicit density reconstruction and can naturally be generalized to multivariate settings. The nearest-neighbor approach to entropy estimation dates back to Kozachenko and Leonenko [10] and was comprehensively studied thereafter in [11,12,13,14,15,16]. Regarding divergence estimation, kNN-based estimators and their consistency properties have been improved in [17,18,19,20], among others.

In addition to R’enyi entropy and divergence, Cauchy–Schwarz (CS) divergence has also been used to develop information-theory-based goodness-of-fit procedures. Since R’enyi-based approaches use entropy functionals and nearest-neighbor-type estimators in multivariate problems, they are well-suited to the current context [12,21]. Methods based on CS divergence offer another non-parametric alternative, typically associated with kernel or Parzen window estimation [22]. This article, however, emphasizes the case of Shannon entropy/KL divergence, where the Gaussian distribution satisfies the natural maximum entropy criterion under fixed mean and covariance constraints.

This paper, building upon this line of work, focuses on the nearest neighbor estimators of KL divergence in a multivariate continuous setting and highlights the goodness-of-fit test. The innovative aspect of this study is not the proposal of a new kNN entropy estimator but rather the use of this estimator in a KL-based entropy difference test for multivariate normality.

The main contributions can be summarized as follows:

- We clarify how the Gaussian maximum-entropy principle leads to an entropy-gap representation of the KL divergence under fixed mean and covariance constraints.
- We constructed a KL-based multivariate normality statistic by combining the moment-matched Gaussian benchmark with the kNN Shannon entropy estimator.
- We calibrated the proposed statistic by parametric bootstrap and studied its finite-sample behavior through Monte Carlo experiments, including the effects of dimension, sample size, and the nearest-neighbor parameter (k).

The rest of the article has been organized as follows. Section 2 restates the principles of maximum entropy and describes their application to KL divergence and Gaussian benchmarks. Section 3 introduces the nearest neighbor estimators of Shannon entropy and the obtained KL divergence estimators. Section 4 provides a framework for hypothesis testing. Section 5 describes the numerical experiments, and Section 6 briefly discusses possible extensions before concluding.

## 2. Maximum Entropy Principles

The principle of maximum entropy, proposed by Jaynes [4], indicates that among all probability distributions consistent with a particular set of constraints (usually moment conditions), that distribution with the greatest entropy is the least informative choice. Because KL divergence and Shannon entropy are closely related, expressions for maximum entropy can often be rewritten in terms of minimum KL divergence relative to a suitable comparison distribution.

First of all, it is useful to formalize the class of acceptable solutions within the standard mean-covariance setting.

**Definition 1**  (Class K). *The distribution class H(f) of density functions f on $R^{m}$ with finite and moment constraints*

$$\begin{array}{cc} \int_{R^{m}}xf\left(x\right)\;dx & =\mu ,\\ \int_{R^{m}}\left(x-\mu \right){\left(x-\mu \right)}^{⊤}f\left(x\right)\;dx\; & =\Sigma , \end{array}$$

*where $\mu \in R^{m}$ denotes a mean vector and Σ denotes a symmetric positive definite covariance matrix of size m×m.*

In the class K, it is well known that the multivariate Gaussian distribution with mean μ and covariance Σ maximizes Shannon entropy; see, for example, [3,5,6]. Specifically, for any f∈K,

(4) $$H\left(f\right)\leq \frac{1}{2}\log\left[{\left(2\pi e\right)}^{m}\det\left(\Sigma \right)\right],$$

and the equality applies if and only if *f* is a Gaussian density.

(5) $$\begin{array}{c} \phi_{\mu ,\Sigma }\left(x\right)=\frac{1}{{\left(2\pi \right)}^{m/2}\det{\left(\Sigma \right)}^{1/2}}\exp\;\left(-\frac{1}{2}{\left(x-\mu \right)}^{⊤}\Sigma^{-1}\left(x-\mu \right)\right). \end{array}$$

The following proposition rephrases this classical result of maximum entropy in KL divergence terms and will serve simply as a baseline for our testing procedures.

**Proposition 1**  (KL divergence Gauss benchmark). *Assume that K is defined as in Definition 1 and $\phi_{\mu ,\Sigma }$ represents a Gaussian density (5), with mean μ and covariance Σ. Then, for every f∈K,*

(6) $$D_{KL}\left(f\;∥\;\phi_{\mu ,\Sigma }\right)=H\left(\phi_{\mu ,\Sigma }\right)-H\left(f\right)\;\geq \;0,$$

*with equality if and only if $f=\phi_{\mu ,\Sigma }$ holds almost everywhere (a.e.).*

The result arises from the maximum entropy property of the Gaussian distribution under the constraints of a fixed mean and covariance; see, for example, [3,5,6].

Proposition 1 suggests that the principle of maximum entropy can be interpreted as the minimum KL divergence principle in the constrained class K: among all densities f∈K with mean μ and covariance Σ, there is a unique maximum value of entropy for the Gaussian $\phi_{\mu ,\Sigma }$ and, equivalently, the KL divergence $D_{KL}\left(f∥\phi_{\mu ,\Sigma }\right)$ measures the entropy gap of *f* relative to this maximum. Specifically, then for any f∈K,

$$D_{KL}\left(f\;∥\;\phi_{\mu ,\Sigma }\right)=0\;⟺\;f=\phi_{\mu ,\Sigma }\;a.e.,$$

That justifies using Gaussian models as a benchmark for testing goodness-of-fit and model validation.

Although similar maximum entropy formulations have been developed for other test families, we focus in this paper on the Gaussian test defined by fixed mean and covariance constraints. For the isotropic case, suppose *f* is the density of $X\in R^{m}$ supported on $R^{m}$ and assume that for some s>0 the moment ${E∥X∥}^{s}$ is finite. Results such as [15,23,24] yield entropy inequalities of the following form

$$H\left(f\right)\leq m\log\;\left({\alpha \left(m,s\right)\;E∥X∥}^{s}\right),$$

which holds for an explicit constant α(m,s) associated with *m* and *s* if and only if *X* satisfies the isotropic generalized Gaussian distribution $GG_{\tau }\left(m,s\right)$ for the corresponding scale parameter τ. The same statement holds for multivariate exponential power distributions with general location and variance matrices; see, e.g., [15,24]. For these classes, the corresponding constraint is formulated in terms of radial moments instead of second-order moments. As in Proposition 1, these entropy bounds can be reformulated as KL inequalities associated with the corresponding generalized Gaussian benchmark $g_{GG}$. For the case where *f* and $g_{GG}$ share the same constraint (e.g., the same ${E∥X∥}^{s}$ in an isotropic medium), we have the following result

$$D_{KL}\left(f\;∥\;g_{GG}\right)=H\left(g_{GG}\right)-H\left(f\right)\;\geq \;0,$$

and the equality is valid if and only if $f=g_{GG}$ almost everywhere. From this point of view, KL-based tests can also be constructed with non-Gaussian maximum entropy benchmarks; in this paper, however, we restrict our attention to the classical Gaussian mean-covariance case.

## 3. Nearest-Neighbor Estimators of the Shannon Entropy and KL Divergence

Estimating Shannon entropy and the KL divergence is a crucial step in many nonparametric inference problems. For multivariate settings, the nearest neighbor methods provide a practical alternative to histogram and kernel-based estimators, in particular, when dimension *m* is moderate and explicit multivariate density estimation has become unstable.

We recall the *k*-nearest neighbor (kNN) estimator of Shannon entropy, describe how it leads to the KL divergence estimator, and outline the asymptotic properties that form the basis of our testing procedure.

### 3.1. Nearest-Neighbor Estimation of Shannon Entropy

Suppose $X_{1},\ldots ,X_{N}$ are i.i.d. random vectors in $R^{m}$ with density *f* and $Y_{1},\ldots ,Y_{M}$ are i.i.d. random vectors in $R^{m}$ with density *g*, which are independent of the *X* sample. Let us assume that *f* and *g* are (Lebesgue) densities which are almost everywhere continuous and almost everywhere positive on their supports and satisfy the regularity and tail/moment conditions specified in Section 3 when specifying asymptotic results.

The nearest neighbor estimator of Shannon entropy was introduced by Kozachenko and Leonenko [10], reviewed in more detail in [11,12], and it is based on a geometric approach to local density: The sample $X_{i}$ becomes sparse around $X_{i}$ when its *k*th nearest neighbor is distant, and the principal density decreases; when neighbors are close, the principal density increases.

We assume that $\rho_{i,k,N}$ denotes the Euclidean distance from $X_{i}$ to its *k*th nearest neighbor among ${\left\{X_{j}\right\}}_{j\neq i}$. The standard version of the Kozachenko–Leonenko (kNN) estimator of Shannon entropy is the following

(7) $${\hat{H}}_{N,k}\left(f\right)=\psi \left(N\right)-\psi \left(k\right)+\log V_{m}+\frac{m}{N}\sum_{i=1}^{N}\log\rho_{i,k,N},$$

where $V_{m}=\pi^{m/2}/\Gamma \left(\frac{m}{2}+1\right)$ is the volume of the unit ball in $R^{m}$ and ψ(·) is the digamma function.

The estimator can be seen as an extension of the statement below:

$$H\left(f\right)=-E[\log f\left(X_{1}\right)],$$

where we replace $f(X_{i})$ with the local kNN density approximation guided by $\rho_{i,k,N}$. Adjustment parameter *k* balances bias and variance: a small *k* results in lower bias but higher variance, whereas a larger *k* increases bias to stabilize the estimate. In practice, *k* is typically fixed to an integer (e.g., k∈{3,5,10}, consistent with *k* constant asymptotic theory and sufficient for the dimensions considered in our experiments.

**Remark 1.** 
*Some equivalent presentations include the constants in a single logarithm, for example,*

$${\hat{H}}_{N,k}\left(f\right)=\frac{1}{N}\sum_{i=1}^{N}\log\;\left(\rho_{i,k,N}^{m}V_{m}e^{\psi (N)-\psi (k)}\right),$$

*and uses ψ(N)≈log(N−1) for large N. As it is standard in the theoretical literature and makes the finite sample constants explicit, we preserve the digamma form in (7).*

### 3.2. Nearest-Neighbor Estimation of KL Divergence

To estimate $D_{KL}\left(f∥g\right)$, a direct additive approach estimates *f* and *g* separately and then combines these estimates using (1). This strategy may acquire the sensitivity of tuning parameters and the high variations in finite samples characteristic of direct density estimation [7,8,9]. Let $X_{1},\ldots ,X_{N}∼f$ and $Y_{1},\ldots ,Y_{M}∼g$ be independent samples, and let $\nu_{i,k}$ denotes the Euclidean distance from $X_{i}$ to its *k*th nearest neighbor in the *Y* sample. According to [12,17,18], a commonly used kNN estimator for $D_{KL}\left(f∥g\right)$ is

(8) $${\hat{D}}_{KL}\left(f∥g\right)=\frac{m}{N}\sum_{i=1}^{N}\log\frac{\nu_{i,k}}{\rho_{i,k,N}}+\psi \left(M\right)-\psi \left(N-1\right).$$

Statistics compare the typical neighbor sizes around each $X_{i}$ when neighbors are obtained from the same distribution (via $\rho_{i,k,N}$) and from the second distribution (via $\nu_{i,k}$). When *f* and *g* are closely related, these radii are comparable, and the estimated deviation is small; systematic differences produce a positive estimate. Intuitively, (8) follows from the following identity

$$D_{KL}\left(f∥g\right)=E\left[\log f\left(X_{1}\right)\right]-E\left[\log g\left(X_{1}\right)\right],$$

where each log-density is substituted with a kNN-based approximation constructed from the associated sample. In the difference, the volume term $V_{m}$ cancels out, which explains its absence in (8). For large M,N values, $\psi \left(M\right)-\psi \left(N-1\right)\approx \log\;\left(M/(N-1)\right)$ can be employed, but we retain the digamma form to follow the standard bias correction.

### 3.3. Asymptotic Properties

There is a well-developed asymptotic theory for kNN entropy and divergence estimators; see, among others, [10,11,12,13,14,15,18,21]. For our objectives, it suffices to provide a representative consistency conclusion for a fixed *k* under standard smoothness and tail conditions.

**Assumption 1** 
(Regularity conditions for the kNN estimators). *In (f) and (g), the densities are continuous, bounded, and positive almost everywhere on the support sets. Furthermore, (H(f)), (H(g)), and (D_KL(f|g)) are finite, and for some δ>0, ${E|X|}^{m+\delta }<\infty$ and ${E|Y|}^{m+\delta }<\infty$. The nearest-neighbor parameter (k) converges when N,M→∞.*

**Theorem 1** 
(Consistency of kNN estimators). *Under Assumption 1. Suppose that f and g are densities on $R^{m}$ and that H(f) and H(g) are finite. Assume that f and g are almost everywhere continuous, upper bounded, and satisfy the following moment/tail condition We assume that they satisfy a moment/tail condition of the form ${E∥X∥}^{m+\delta }<\infty$ and ${E∥Y∥}^{m+\delta }<\infty$, where X∼f and Y∼g. Let k≥1 be a constant. Then, as N,M→∞,*

$${\hat{H}}_{N,k}\left(f\right)\overset{a.s.}{\to }H\left(f\right),\;{\hat{D}}_{KL}\left(f∥g\right)\overset{a.s.}{\to }D_{KL}\left(f∥g\right).$$

*Under the same conditions, the estimators will also be consistent in terms of the mean square error, i.e., $\mathrm{Var}({\hat{H}}_{N,k}\left(f\right))\to 0$ and $\mathrm{Var}({\hat{D}}_{KL}\left(f∥g\right))\to 0$, and will be asymptotically unbiased.*

The above statement is consistent with the $L^{2}$-consistency framework introduced in [13,14]. In particular, Cadirci et al. [21] employ Poissonization techniques to estimate the mean square convergence of the Kozachenko–Leonenko entropy estimator for a random fixed k≥1 in the context of entropy-based testing. A similar set of techniques, together with the unbiased form (8), supports the associated mean square consistency claims for the kNN KL estimator used here.

**Remark 2.** 
*Depending on whether the support is bounded or unbounded, different sources specify slightly different sufficiency conditions. In the case of bounded support, it is generally assumed that there is a positive lower bound on the density within the support; in the case of unbounded support (including Gauss and related families), conditions on moments and tails substitute for global lower bounds. In our numerical experiments, we chose the conditions in Theorem 1 to cover the distributions used, and they remain close to the standard assumptions in the cited references.*

In summary, the nearest neighbor methods offer a simple and theoretically grounded way to estimate both Shannon entropy and KL divergence in multivariate problems. These approaches do not require explicit multivariate density reconstruction and are convenient to apply for the sample sizes and dimensions discussed in this study; the computational cost has been specified in the numerical section.

## 4. Goodness-of-Fit Tests Based on KL Divergence

Based on the KL divergence, we performed a goodness-of-fit test for multivariate normality. Let $X_{1},\ldots ,X_{N}$ be i.i.d. random vectors in $R^{m}$ with density f, finite mean μ=E[X], and covariance Σ=Var(X) as i.i.d random vectors in $R^{m}$. We tested the composite null hypothesis

$$H_{0}:\;f\in F_{N}\;(\mathrm{family}\;\mathrm{of}\;m-\mathrm{variable}\;\mathrm{Gaussians}\;\mathrm{with}\;\mathrm{unconditional}\;\mathrm{mean}\;\mathrm{and}\;\mathrm{covariance})$$

against the general alternative $H_{1}:\;f\notin F_{N}$.

Let

$${\bar{X}}_{N}=\frac{1}{N}\sum_{i=1}^{N}X_{i},\;S_{N}=\frac{1}{N-1}\sum_{i=1}^{N}\left(X_{i}-{\bar{X}}_{N}\right){\left(X_{i}-{\bar{X}}_{N}\right)}^{⊤}$$

be the sample mean and sample covariance matrix, respectively, and suppose that $\phi_{{\bar{X}}_{N},S_{N}}$ is the Gaussian density having this mean and covariance. We recall from Proposition 1 that, within the class K of distributions with fixed mean μ and covariance Σ, the multivariate Gaussian density $\phi_{\mu ,\Sigma }$ uniquely maximizes the Shannon entropy and satisfies

$$D_{KL}\left(f∥\phi_{\mu ,\Sigma }\right)=H\left(\phi_{\mu ,\Sigma }\right)-H\left(f\right)\geq 0,$$

with equality if and only if $f=\phi_{\mu ,\Sigma }$ almost everywhere and

$$H\left(\phi_{\mu ,\Sigma }\right)=\frac{1}{2}\log\left[{\left(2\pi e\right)}^{m}\det\left(\Sigma \right)\right].$$

Based on this idea, let us define the KL-based test statistic

(9) $$T_{N,k}^{\mathrm{KL}}:=H\;(\phi_{{\bar{X}}_{N},S_{N}})-{\hat{H}}_{N,k}\left(f\right)=\frac{1}{2}\log\left[{\left(2\pi e\right)}^{m}\det\left(S_{N}\right)\right]-{\hat{H}}_{N,k}\left(f\right),$$

where ${\hat{H}}_{N,k}\left(f\right)$ is an estimator of the Shannon entropy given in (7) using the kNN. As constructed, $T_{N,k}^{\mathrm{KL}}$ gives an estimate of the KL divergence $D_{KL}\left(f∥\phi_{\mu ,\Sigma }\right)$ using the identity $D_{KL}\left(f∥g\right)=H\left(g\right)-H\left(f\right)$.

**Theorem 2.**  *Suppose that f is a continuous density on $R^{m}$ with finite second moments and that the conditions of Theorem 1 are satisfied. For any fixed k≥1,*

$$T_{N,k}^{\mathrm{KL}}\overset{p}{\to }H\left(\phi_{\mu ,\Sigma }\right)-H\left(f\right)=D_{KL}\left(f∥\phi_{\mu ,\Sigma }\right)\;\mathrm{as}\;N\to \infty .$$

*In particular,*

$$T_{N,k}^{\mathrm{KL}}\overset{p}{\to }0\;\mathrm{if}\;X∼N_{m}\left(\mu ,\Sigma \right),$$

*and $T_{N,k}^{\mathrm{KL}}\overset{p}{\to }\xi \left(f\right)>0$ for any non-Gaussian f∈K having the same mean μ and covariance Σ.*

**Proof.**  We decompose

$$T_{N,k}^{\mathrm{KL}}=\underset{\left(I\right)}{\underset{︸}{\left(H\left(\phi_{{\bar{X}}_{N},S_{N}}\right)-H\left(\phi_{\mu ,\Sigma }\right)\right)}}+\underset{\left(\mathrm{II}\right)}{\underset{︸}{\left(H\left(\phi_{\mu ,\Sigma }\right)-H\left(f\right)\right)}}+\underset{\left(\mathrm{III}\right)}{\underset{︸}{\left(H\left(f\right)-{\hat{H}}_{N,k}\left(f\right)\right)}}.$$

By the Law of Large Numbers, $S_{N}\to \Sigma$ almost surely, hence (I) →0, since N→∞ due to the continuous nature of logdet(·) on the set of positive definite matrices. Under Proposition 1, term (II) equals $D_{KL}\left(f∥\phi_{\mu ,\Sigma }\right)$ and vanishes to zero in probability under the assumptions of Theorem 1.

According to the law of large numbers, ${\bar{X}}_{N}\to \mu$ and $S_{N}\to \Sigma$ in probability. Considering that Σ is defined as positive, the stability of logdet(·) suggests that

$$H\left(\phi_{{\bar{X}}_{N},S_{N}}\right)\to H\left(\phi_{\mu ,\Sigma }\right).$$

Furthermore, under Assumption 1, we have

$${\hat{H}}_{N,k}\left(f\right)\to H\left(f\right).$$

Therefore,

$$T_{N,k}^{\mathrm{KL}}\to H\left(\phi_{\mu ,\Sigma }\right)-H\left(f\right)=D_{KL}\left(f∥\phi_{\mu ,\Sigma }\right).$$

This limit is zero with the Gaussian null hypothesis, whereas it is strictly positive for non-Gaussian alternatives with the same mean and covariance, by the maximum entropy property.    □

Practically, it is not possible to obtain the zero distribution of $T_{N,k}^{\mathrm{KL}}$ in closed form. We therefore use parametric bootstrapping with an adapted Gaussian model to calibrate the test. With $N_{m}\left({\bar{X}}_{N},S_{N}\right)$, we performed the calibration using parametric bootstrap. We simulated bootstrap samples for the selected neighborhood size *k* and significance level α∈(0,1) $X_{1:N}^{\left(b\right)}∼N_{m}\left({\bar{X}}_{N},S_{N}\right)$, calculated $T_{N,k}^{\mathrm{KL}}$ for each sample, and took the (1−α)-quantile of the resulted bootstrap values as the critical threshold $t_{\alpha }$. When $T_{N,k}^{\mathrm{KL},\mathrm{obs}}\geq t_{\alpha }$, we reject $H_{0}$.

**Remark 3**  (KL divergence as expected log-loss). *The divergence*

$$D_{KL}\left(f∥\phi_{\mu ,\Sigma }\right)=\int_{R^{m}}f\left(x\right)\log\;\frac{f(x)}{\phi_{\mu ,\Sigma }\left(x\right)}\;dx$$

*is interpreted as the average log-likelihood loss incurred by employing the Gaussian model $\phi_{\mu ,\Sigma }$ to substitute the true density f. In fact,*

$$D_{KL}\left(f∥\phi_{\mu ,\Sigma }\right)=-\;E_{f}\left[\log\phi_{\mu ,\Sigma }\left(X\right)\right]+H\left(f\right),$$

*which means it measures the excess cross-entropy (equivalently, it is the increase in expected log-loss) relative to an oracle model that knows f.*

Since analytical null distributions for $T_{N,k}^{\mathrm{KL}}$ are difficult to obtain, we calibrated critical values using parametric bootstrap with $H_{0}$ (Algorithm 1), by following related entropy- and KL-based goodness-of-fit tests in [25,26,27,28,29].
**Algorithm 1** Bootstrap calibration for $T_{N,k}^{\mathrm{KL}}$1:**Input:** data $X_{1:N}$, number of bootstrap replications *B*, significance level α, neighborhood size *k*.2:Calculate ${\bar{X}}_{N}$ and $S_{N}$.3:Calculate $T_{N,k}^{\mathrm{KL},\mathrm{obs}}$ from $X_{1:N}$ by using (9).4:**for** b=1,…,B **do**5:       Simulate a bootstrap sample of size *N*: $X_{1:N}^{\left(b\right)}∼N_{m}\left({\bar{X}}_{N},S_{N}\right)$.6:       Calculate $T_{N,k}^{\mathrm{KL},(b)}=T_{N,k}^{\mathrm{KL}}\left(X_{1:N}^{\left(b\right)}\right)$.7:**end for**8:Let $t_{\alpha }$ be the (1−α)-quantile of ${\left\{T_{N,k}^{\mathrm{KL},(b)}\right\}}_{b=1}^{B}$.9:**Decision:** reject $H_{0}$ if $T_{N,k}^{\mathrm{KL},\mathrm{obs}}\geq t_{\alpha }$.

## 5. Numerical Experiments

In this section, numerical experiments are reported for the KL-based statistic $T_{N,k}^{\mathrm{KL}}$ specified in (9). There are three objectives. We first display the finite sample behavior of the statistic in terms of sample size *N*, dimension *m*, and neighborhood size *k*. Secondly, the empirical power is examined against structured non-Gaussian alternatives, including light and heavy tailed deviations. Finally, in practical applications, we provide calibrated 5% critical values. As noted, Monte Carlo results are based on independent repetitions, and error bars are based on an empirical standard deviation. For all experiments, we used Euclidean distances in the *k*-nearest neighbor calculations. All simulations were performed using Python 3.10, and with the reported simulation settings, a single full run calibrated using bootstrap took approximately 60–90 s.

### 5.1. Monte Carlo Study of $T_{N,k}^{\mathrm{KL}}$: Convergence and Stability

We examine the convergence behavior of $T_{N,k}^{\mathrm{KL}}$ first. Proposition 1 implies that, under multivariate normality conditions, the population target is $D_{KL}\left(f∥\phi_{\mu ,\Sigma }\right)=0$, and therefore the statistic should converge to zero as *N* increases. To evaluate the robustness beyond the Gaussian case, we also simulated examples from generalized Gaussian models having shape parameter *s*, noting that s=2 corresponds to the Gaussian reference value in this family.

**Figure 1:**
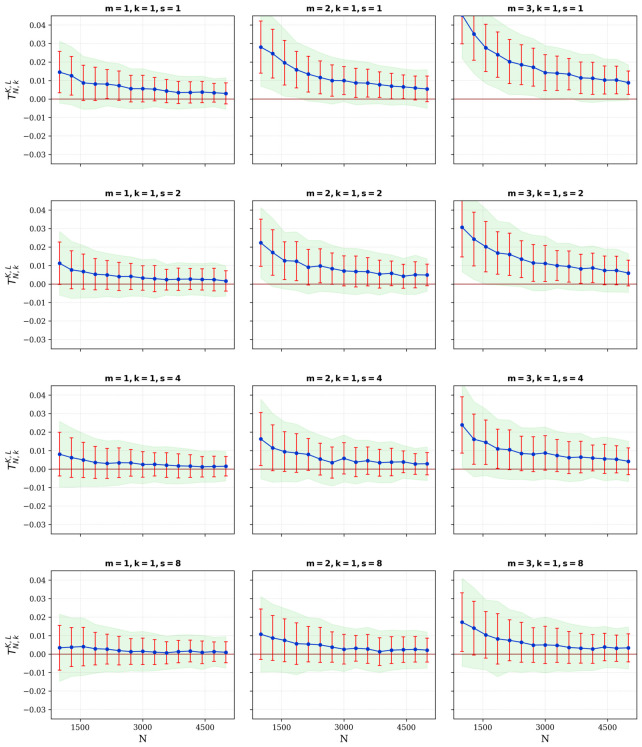
Convergence behavior of $T_{N,k}^{\mathrm{KL}}$ for the neighborhood dimension k=1. Error bars represent one standard deviation empirically over M=100 iterations. This corresponds to the Gaussian benchmark, s=2; here, the statistic becomes concentrated near zero as *N* increases.

We conducted (M=100) independent repetitions of this descriptive convergence experiment. This experiment was used to demonstrate that the statistic is qualitatively stable; however, it is the main power and critical value analyses described below that are supported by (M=1000) repetitions. Figure 1 reports the results for N∈{1000,…,5000}. In the Gaussian case (s=2), the statistic approaches zero. For s≠2, the statistic stabilizes away from zero, consistent with convergence to a positive KL bound with fixed non-Gaussian alternatives.

**Figure 2:**
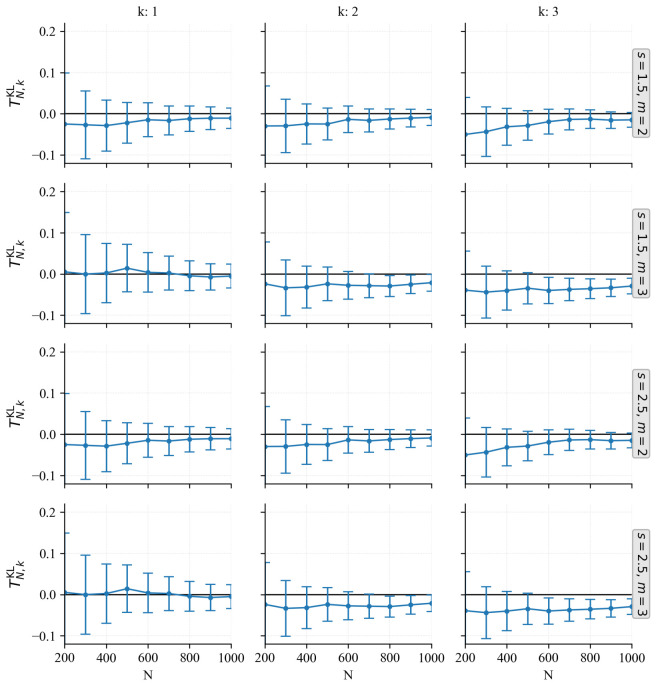
Finite sample stability of $T_{N,k}^{\mathrm{KL}}$ over neighborhood dimensions k∈{1,2,3}. Error bars indicate one empirical standard deviation. Variability decreases with *N* and further decreases with larger neighborhoods.

The overlapping error bars suggest that small differences between neighboring curves should not be overinterpreted. Consequently, we mainly use the figure to illustrate the general trend of $T_{N,k}^{\mathrm{KL}}$ as *N* increases. Subsequently, we repeated the experiment for smaller sample sizes N∈{100,…,1000} and k∈{1,2,3} to investigate the finite sample regime and the effect of *k*. Figure 2 illustrates that increasing *k* reduces variability essentially without changing the central tendency. These results are consistent with the standard bias–variance trade-off in kNN functionals: larger neighborhoods provide an additional mean and therefore provide a smaller variance, but this usually comes at the cost of a modest increase in bias [15]. In the current (m,N) range, variance decrease is the dominant and visible effect.

Parameter (k) controls the typical bias–variance trade-off of nearest-neighbor estimators. Larger values of (k) decrease variability by using averages over wider neighborhoods; however, they may also result in a smoothing bias. For this reason, (k) may be considered as a tuning parameter. For practical applications, (k) can be determined based on (K)-fold cross-validation or a bootstrap stability criterion applied to a predefined candidate set. To demonstrate the sensitivity of the proposed statistic, several fixed (k) values are reported in this study.

### 5.2. Power of the Goodness-of-Fit Test

For each configuration, we computed the bootstrap critical value utilizing Algorithm 1 using the fitted Gaussian model $N_{m}\left({\bar{X}}_{N},S_{N}\right)$. We evaluate empirical power for testing multivariate normality at the significance level α=0.05. In this case, the empirical power is the proportion of Monte Carlo replications (generated with the alternative hypothesis) for which the observed $T_{N,k}^{\mathrm{KL}}$ exceeds the bootstrap critical value.

In power experiments, M=1000 replications and sample sizes N∈{500,1000} were used. We considered two complementary classes of alternatives: (i) deviations within a generalized Gaussian family (controlled tail behavior) and (ii) heavy-tailed Student-type alternatives (departures outside the generalized Gaussian family for finite degrees of freedom).

#### 5.2.1. Power Against Generalized Gaussian Alternatives

**Figure 3:**
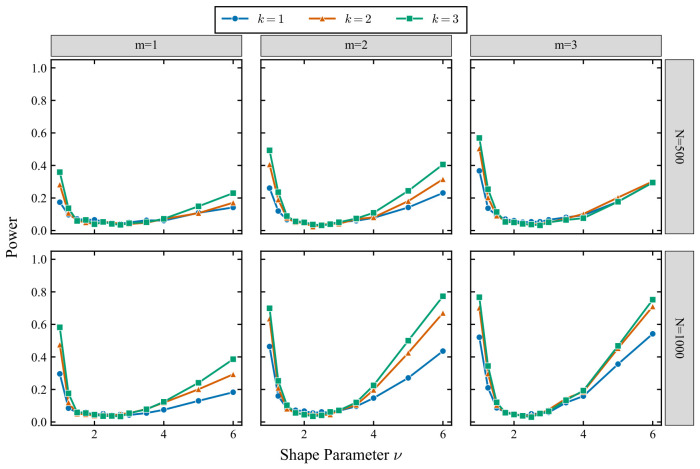
Empirical power of $T_{N,k}^{\mathrm{KL}}$ against generalized Gaussian alternatives. Curves compare (k∈1,2,3) across dimensions (m=1,2,3); rows correspond to (N=500) and (N=1000), with (M=1000) Monte Carlo replications.

For the first experiment, we generalized the Gaussian family by shifting the shape parameter away from the Gaussian reference value. Specifically, we kept the location and variance fixed and compared the Gaussian case with X∼GG(m,v) where v≠2. Figure 3 displays the resulting power curves.

The partial overlap of the power curves suggests that the contribution of (k) has a moderate effect in some configurations; hence, small differences between closely spaced curves needs to be interpreted with caution. In general, power increases as the deviation from the Gaussian reference increases.

There is consistency between the two model sizes. First, power increases as the deviation from the Gaussian distribution increases, reflecting the consistency of $T_{N,k}^{\mathrm{KL}}$ against fixed alternatives. Second, increasing the sample size from N=500 to N=1000 produces a noticeable increase in sensitivity. Choice of neighborhood size *k* has a secondary but systematic effect: larger neighborhoods often produce smoother and less variable power curves, consistent with the variance reduction often observed in Figure 2.

#### 5.2.2. Power Against Student-Type Alternatives

**Figure 4:**
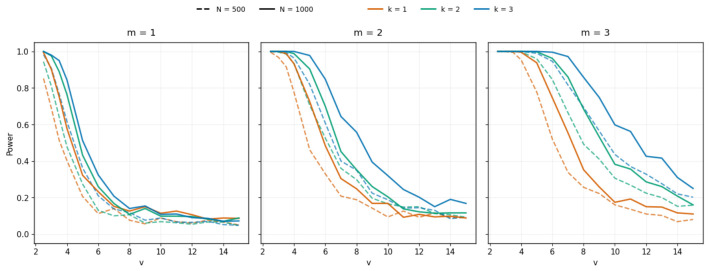
The empirical power of $T_{N,k}^{\mathrm{KL}}$ compared to studentized alternatives. The solid and dashed lines represent N=1000 and N=500, respectively; the lines compare k∈{1,2,3}.

Next, we will consider heavy-tailed Student’s t-type alternatives parameterized by degrees of freedom ν. Here, smaller ν values correspond to heavier tails. Figure 4 illustrates that the power approximates one in heavy-tailed cases (small ν) and decreases as ν increases. It is expected that this trend occurs because larger ν values produce distributions that are increasingly similar to the Gaussian reference, making it harder to distinguish the alternative. For N=500 and N=1000, the distinction between the curves once again confirms that larger sample sizes increase sensitivity. For most configurations, moderate neighborhood sizes produce slightly more stable power profiles, which is consistent with the stability effects observed in convergence experiments.

### 5.3. Empirical Distribution of the Standardized Statistic

**Figure 5:**
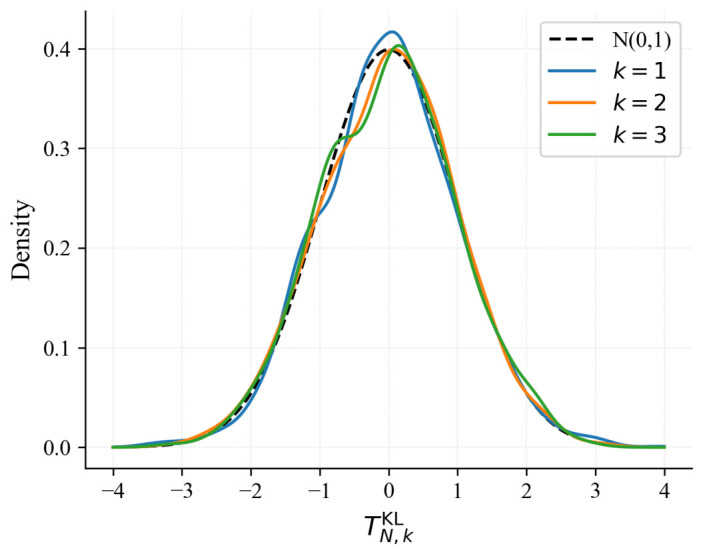
For N=1000 and k∈{1,2,3}, kernel density estimates of the standardized statistic $Z_{N,k}$ were evaluated and compared with the standard Gaussian density N(0,1).

Although calibrated using bootstrap testing, examining the shape of the statistic after standardization can be informative.

$$Z_{N,k}=\frac{T_{N,k}^{\mathrm{KL}}-{\hat{\mu }}_{N,k}}{{\hat{\sigma }}_{N,k}},$$

where ${\hat{\mu }}_{N,k}$ and ${\hat{\sigma }}_{N,k}$ represent the empirical mean and standard deviation computed from Monte Carlo replications using the Gaussian benchmark. Figure 5 contrasts kernel density estimates of $Z_{N,k}$ at N=1000 and k∈{1,2,3} with the standard normal density. In this regime, the fit is close, indicating that the normal approximation may be appropriate for descriptive purposes. Figure 5 has been included as a diagnostic check of the finite-sample behavior of the standardized statistic using the Gaussian reference model. The test is calibrated using parametric bootstrap, so it is not used to determine the rejection rule; however, the figure demonstrates whether the standardized statistic has a shape that is approximately symmetric and similar to a normal distribution in the given setting. To emphasize that this evidence is exploratory, throughout the study, rejection thresholds were computed using the parametric bootstrap within Algorithm 1, which is not based on the assumed asymptotic normal limit.

**Figure 6:**
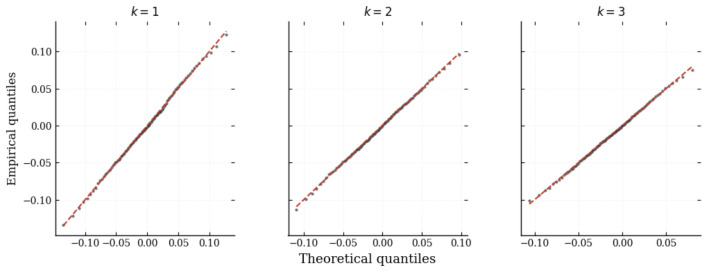
Normally distributed Q-Q plots of the standardized statistic $Z_{N,k}$ for N=1000 and k∈{1,2,3}.

Figure 6 gives a complementary Q-Q plot. Consistent with the KDE evidence, the empirical quantiles closely approximate the reference line, including the tails.

### 5.4. Finite-Sample Rate of Convergence: A Log–Log Regression Diagnostic

To summarize the convergence rate of $T_{N,k}^{\mathrm{KL}}$ with the Gaussian benchmark, we fit a simple log–log regression to the Monte Carlo estimates of $\left|E[T_{N,k}^{\mathrm{KL}}\left(m,s\right)]\right|$ across increasing sample sizes:

(10) $$\log\left|E\left[T_{N,k}^{\mathrm{KL}}\left(m,s\right)\right]\right|=\alpha_{m,s,k}+\beta_{m,s,k}\;\log N.$$

For kNN entropy functionals, the leading bias term is typically of order $O(N^{-1/2})$ in normal regimes. Based on this intuitive approach, in the Gaussian benchmark (corresponding to s=2 in the generalized Gaussian family), $\beta_{m,s,k}\approx -\frac{1}{2}$ is expected. Thus, deviations from this benchmark can be explained as slower decay or stronger finite sample effects.

Table 1 shows estimations of the slopes for dimensions *m*, neighborhood sizes *k*, and shape parameters *s*. These values reach their minimum near the Gaussian reference value s=2, in which case convergence is fastest. The slopes increase slightly but remain small as we move away from s=2, which is consistent with the stable behavior of the statistic for both heavy and light tailed generators. Furthermore, as *k* increases from 1 to 3, we notice a slight decrease in β, reflecting improved stability in the nearest neighbor estimation for larger neighborhoods. In Table 1, the reported regression coefficients complement the log–log diagnostic analysis and illustrate how the prediction error (N) changes over time. The fitted trend demonstrates that the error empirically decreases with the Gaussian reference model and supports the convergence behavior noticed in the simulations.

### 5.5. Critical Values for Practical Implementation

We estimated 5% critical values $t_{0.05}$ by parametric bootstrap with the fitted Gaussian model (Algorithm 1), using M=1000 replications. Table 2 shows these thresholds for sample sizes N∈{100,…,1000}, dimensions m∈{2,3}, and neighborhood sizes k∈{1,2,3}. (Rows indexed by *s* can be understood as a sensitivity check across different generators; for Gaussian calibration, the relevant row is s=2).

Table 1 and Table 2, when considered together, summarize the two complementary aspects of the proposed procedure. Table 1 explains the empirical convergence behavior of the statistic, whereas Table 2 offers finite-sample bootstrap critical values for practical applications. As (N) increases, the decrease in critical values corresponds to the convergence of $(T_{N,k}^{\mathrm{KL}}$) to zero at the Gaussian reference point; however, the larger values for (m=3) correspond to the increasing difficulty of locally estimating entropy in higher-dimensional multivariate settings.

These critical values slowly decrease as *N* increases, reflecting that $T_{N,k}^{\mathrm{KL}}$ becomes close to zero with the Gaussian benchmark. With a fixed *N*, thresholds are larger for m=3 than for m=2, consistent with the increased challenges of local entropy estimation in higher dimensions. Typically, increasing *k* yields slightly smaller thresholds, aligning with the variance reduction illustrated in Figure 2.

In the applications, when the observed statistic $T_{N,k}^{\mathrm{KL}}\geq t_{0.05}$ is satisfied, the multivariate normality is rejected at the α=0.05 level. In this case, $t_{0.05}$ is calculated using bootstrap calibration (Algorithm 1) or obtained from Table 2 for the settings reported here.

## 6. Conclusions

In this paper, a framework based on information theory is proposed for multivariate goodness-of-fit testing, utilizing a kNN entropy estimate and bootstrap calibration, thereby avoiding the explicit reconstruction of multivariate density functions.

We developed a KL-decomposition approach for a multivariate normality test using the k-nearest neighbor estimate of Shannon entropy. The fundamental structural feature is that, within a fixed mean-covariance class, the Gaussian reference value converts the KL decomposition into an entropy difference, such that the statistic $T_{N,k}^{\mathrm{KL}}$ predicts only a non-negative inconsistency that vanishes under multivariate normality conditions. Under standard regularity conditions, the kNN entropy estimator is consistent, which directly translates to the consistency of the resulting KL-based test statistic.

Our numerical study provides three practical results. First, statistics in the Gaussian benchmark rapidly approach zero as *N* increases. Secondly, the power increases smoothly with deviation power, including shape changes in generalized Gaussian models and heavy-tailed Student-type alternatives. Finally, bootstrap calibration provides stable finite sample control and generates critical values that behave as predicted by *N*, *m* and *k*.

In future studies, the proposed predictor could be compared in greater detail with other existing goodness-of-fit and deviation-based methods as part of a dedicated simulation study.

## Figures and Tables

**Table 1:** Estimated slope coefficients β from the log–log regression (10) for $\left|E[T_{N,k}^{\mathrm{KL}}\left(m,s\right)]\right|$ against *N*.

*s*	m=1			m=2			m=3		
	k=1	k=2	k=3	k=1	k=2	k=3	k=1	k=2	k=3
1.0	0.0092	0.0085	0.0078	0.0124	0.0110	0.0095	0.0145	0.0131	0.0118
1.5	0.0041	0.0035	0.0029	0.0058	0.0049	0.0042	0.0072	0.0064	0.0056
2.0	0.0004	0.0002	0.0001	0.0008	0.0005	0.0003	0.0012	0.0009	0.0007
2.5	0.0006	0.0004	0.0002	0.0011	0.0009	0.0007	0.0018	0.0015	0.0012
3.0	0.0015	0.0012	0.0009	0.0024	0.0020	0.0017	0.0031	0.0028	0.0024
4.0	0.0032	0.0028	0.0023	0.0045	0.0039	0.0034	0.0056	0.0051	0.0046

**Table 2:** Statistics for KL-based tests $T_{N,k}^{\mathrm{KL}}\left(m,s\right)$ critical values $t_{0.05}$ at the 5% level were estimated using M=1000 repeated parametric bootstrap. For Gaussian goodness-of-fit, row s=2 corresponds to the Gaussian reference value.

*s*	*N*	m=2			m=3		
		k=1	k=2	k=3	k=1	k=2	k=3
1.5	100	0.1428	0.1315	0.1254	0.1652	0.1540	0.1488
	200	0.0985	0.0912	0.0865	0.1187	0.1095	0.1052
	300	0.0789	0.0724	0.0691	0.0954	0.0881	0.0843
	400	0.0672	0.0615	0.0588	0.0815	0.0748	0.0712
	500	0.0594	0.0541	0.0519	0.0721	0.0665	0.0634
	600	0.0538	0.0489	0.0467	0.0652	0.0598	0.0571
	700	0.0495	0.0448	0.0426	0.0598	0.0549	0.0525
	800	0.0458	0.0415	0.0395	0.0556	0.0508	0.0485
	900	0.0429	0.0388	0.0368	0.0521	0.0475	0.0452
	1000	0.0405	0.0365	0.0346	0.0492	0.0448	0.0426
2.0	100	0.1256	0.1148	0.1085	0.1485	0.1362	0.1295
	200	0.0864	0.0792	0.0745	0.1054	0.0968	0.0915
	300	0.0695	0.0632	0.0598	0.0856	0.0785	0.0742
	400	0.0592	0.0538	0.0505	0.0728	0.0665	0.0628
	500	0.0524	0.0475	0.0448	0.0645	0.0589	0.0556
	600	0.0475	0.0428	0.0402	0.0582	0.0532	0.0501
	700	0.0436	0.0392	0.0368	0.0535	0.0488	0.0462
	800	0.0405	0.0365	0.0342	0.0495	0.0452	0.0428
	900	0.0378	0.0340	0.0318	0.0464	0.0422	0.0398
	1000	0.0356	0.0319	0.0299	0.0438	0.0398	0.0375
2.5	100	0.1345	0.1228	0.1165	0.1568	0.1452	0.1385
	200	0.0925	0.0856	0.0805	0.1124	0.1028	0.0982
	300	0.0742	0.0678	0.0642	0.0905	0.0825	0.0788
	400	0.0635	0.0575	0.0545	0.0772	0.0705	0.0668
	500	0.0562	0.0508	0.0482	0.0685	0.0622	0.0589
	600	0.0508	0.0458	0.0435	0.0618	0.0562	0.0532
	700	0.0468	0.0420	0.0398	0.0568	0.0515	0.0488
	800	0.0435	0.0390	0.0368	0.0526	0.0478	0.0452
	900	0.0408	0.0365	0.0345	0.0492	0.0448	0.0422
	1000	0.0385	0.0342	0.0324	0.0465	0.0422	0.0398

## Data Availability

The data and code supporting the reported results are publicly available at https://github.com/mehmetsiddik/KL-Divergence.git (accessed on 21 June 2026).
